# Feasibility and implementation of a final year Objective Structured Clinical Examination (OSCE) in a medical model curriculum

**DOI:** 10.1186/s12909-026-10350-3

**Published:** 2026-09-16

**Authors:** Christoph Noll, Marie Mikuteit, Sina Golon, Christian Louzek, Kambiz Afshar, Ingo Just, Sandra Steffens

**Affiliations:** 1 Department of Medical Education, Hannover Medical School, Carl-Neuberg-Strasse 1, Hannover, D-30625 Germany; 2 Department of Dermatology and Allgery, Hannover Medical School, Carl-Neuberg-Straße 1, D-30625, Hannover, Germany; 3 Dean’s office, Hannover Medical School, Hannover, Germany; 4 Institute for General Practice and Palliative Care, Hannover Medical School, Hannover, Germany

**Keywords:** Objective Structured Clinical Examination (OSCE), Final Year OSCE, Development and evaluation of OSCE

## Abstract

**Background:**

Practical clinical skills cannot be adequately assessed through written examinations. This article describes the development and implementation of a summative Objective Structured Clinical Examination (OSCE) prior to the Practical Year (PJ) at Hannover Medical School.

**Methods:**

An interdisciplinary team, medical experts and students developed 34 OSCE stations based on Entrustable Professional Activities. Stations were piloted in 2022 with voluntary student participants and focus group feedback. The PJ-OSCE was curricularly integrated in 2023/24 and linked to five core clinical clerkships/modules. Pass–fail cut scores were established using the modified Angoff method.

**Results:**

The pilot OSCEs proved high staffing demands but overall feasible and favorable for student learning. In June 2024, 278 students completed the mandatory PJ-OSCE across 15 circuits of eight stations (8 min each). Mean score was 603.08 (SD 67.57)/800 points (75.2%); pass rate was 97.1%. Examination reliability averaged 0.84 (Cronbach’s alpha, SD 0.06). Students rated the examination 10.90/15 points, with 41.0% reporting substantial learning gains. Examiners rated the format as appropriate (86.3%).

**Conclusions:**

This feasibility and implementation study demonstrates that a large-scale summative PJ-OSCE can be successfully developed and delivered within a German model curriculum context. The structured development process may serve as a model for other medical faculties implementing similar examination formats.

## Background

During the final year of medical education, the Practical Year (*Praktisches Jahr*, PJ), essential medical competencies—such as communication skills, diagnostic reasoning, clinical decision-making, and interprofessional collaboration—are intended to be further developed. These competencies are reflected in the National Competency-Based Learning Objectives Catalogue for Medicine (NKLM [1]) as well as in the PJ logbooks of the medical faculties.

In contrast, the written second medical state examination (M2) concludes the first five years of undergraduate medical education in Germany. The examination consists of single-best-answer questions and therefore primarily assesses factual knowledge and applied reasoning. However, practical clinical skills acquired during medical training cannot be adequately assessed in a written examination format.

In 2014, the German Science and Humanities Council recommended expanding the application of oral and practical examination formats, such as the Objective Structured Clinical Examination (OSCE [2]). This examination format has also been explicitly included in the draft revised Medical Licensure Act [3].

In an OSCE, students rotate through multiple stations at which communication skills and clinical decision-making can be assessed using standardized simulated patients and cases. Assessment is based on predefined scoring rubrics, ensuring objective evaluation of student performance [4, 5]. OSCEs in earlier medical training are an established component of undergraduate medical education at German medical faculties; however, due to the considerable personnel and time resources required, a nationwide implementation of an OSCE prior to the PJ has not yet been achieved.

At Hanover Medical School, an OSCE at the end of the fifth year of study was developed to assess the practical skills acquired during the curriculum, clinical clerkships, and elective placements. The stations were designed to reflect complex discipline-specific, communicative, and interprofessional competencies. The development of such a PJ-OSCE required expertise in medical education, examination design, and specialist clinical knowledge.

This expertise is based on many years of experience at Hanover Medical School: The practice- and competency-oriented model curriculum “HannibaL” has been established since the academic year 2005/06. Its primary aim is to enable structured patient contact at an early stage of medical training. For more than 13 years, a curricular OSCE has been conducted at the end of the second year of study within the module “Diagnostic Methods.” Although HannibaL predates the formal EPA framework, its cross-disciplinary longitudinal modules and early, structured patient contact provided a curricular foundation compatible with EPA-oriented competency assessment, on which the PJ-OSCE builds.

The purpose of the PJ-OSCE is to assess students’ readiness to enter the Practical Year by evaluating the practical clinical competencies acquired during the preceding curriculum and clinical clerkships, in line with the EPA framework. This article describes the development, feasibility evaluation, and implementation of a PJ-OSCE at Hannover Medical School, with the aim of assessing whether this complex summative assessment format can be successfully delivered at scale in a German medical faculty context.

## Methods

### Development of the PJ-OSCE

The interdisciplinary project team was supported in the development of the PJ-OSCE by the Institute for Communication and Assessment Research (CARES gGmbH), whose platform is used for the established OSCE at Hannover Medical School (MHH).

As a first step, existing station development templates were revised and adapted to the institutional and curricular requirements of MHH. The OSCE stations were based on the Entrustable Professional Activities (EPAs [6]), defined in the National Competency-Based Learning Objectives Catalogue for Medicine (NKLM [1]). In addition, particular emphasis was placed on physician–patient communication and clinical decision-making. It was also stipulated that at least 50.0% of the stations had to include a practical clinical skill selected from a predefined list (Table 1). The list was developed by the OSCE-Team and based on the learning objectives from the NKLM (Chapter VIII.7 [1]). and of the contributing modules.

**Table 1:** Predefined list of clinical skills to be included in at least 50.0% of the OSCE stations

ECG interpretation	
Interpretation of laboratory findings	
Development of a treatment plan	
Surgical wound management	
Gynecological examination	
Preparation and administration of intravenous infusions	
Intramuscular/subcutaneous injections	
Physical examination	
Interpretation of radiological findings	
Sonography for Trauma (eFAST)	
Administration of blood transfusions	
Issuing prescriptions and certificates of incapacity for work	
Cardiopulmonary resuscitation (CPR)	

Each station was developed by faculty members from different clinical disciplines with clinical clerkships. The stations were subsequently reviewed and revised in structured workshops. Each workshop involved the developers of two stations, students, members of the PJ-OSCE team, and a representative from the standardized patient (SP) program. During these workshops (approximately 120–150 min each), the stations were evaluated and pre-piloted. Following this process, all stations underwent a standard-setting procedure with at least six physicians from different specialties using the modified Angoff method to define individual pass–fail cut scores [7, 8]. The standard-setting took place without SPs or students.

### Feasibility assessment and pilot testing

Two pilot OSCEs with different formats were conducted during the lecture-free period in summer 2022 at the MHH SkillsLAB. The pilot examinations lasted 104 and 140 min, respectively, and consisted of 9 or 12 stations of 10 min each, with a 2-minute transition time between stations.

Participants were recruited voluntarily from fifth- and sixth-year medical students and completed the PJ-OSCE without specific preparatory training. Key faculty stakeholders attended the pilot examinations as observers. Prior to the pilots, examiners and standardized patients (SPs) received targeted training focusing on the newly developed stations.

Following the pilot runs, four focus groups (4–6 participants each, total *n* = 22) with participating students were conducted in October 2022. The discussions were audio-recorded, transcribed using large language model–based transcription software (VoiceAI by *Gesellschaft für wissenschaftliche Datenverarbeitung mbH Göttingen*). Anonymized focus group transcripts were analysed using a generative AI-based approach (ChatGPT-4 by OpenAI, web interface) to support initial thematic clustering, focusing on participants’ general evaluation of the OSCE (e.g., feasibility, difficulty, relevance, structure) while excluding statements referring solely to feedback quality. Semantically related statements were inductively grouped into thematic categories and counted at the interview-group level (i.e., number of groups raising a theme, not individual utterances), to avoid over-weighting larger or more talkative groups.

Three researchers reviewed AI-generated themes against the raw German transcripts. This review was sequential rather than blinded; the first author made final theme classifications based on the collective notes, applying a low threshold for inclusion (any explicit mention sufficed). Exact prompt-level parameters from the original analysis were not systematically recorded and are therefore not reported.

### Curricular implementation and examination procedure

**Fig. 1:**
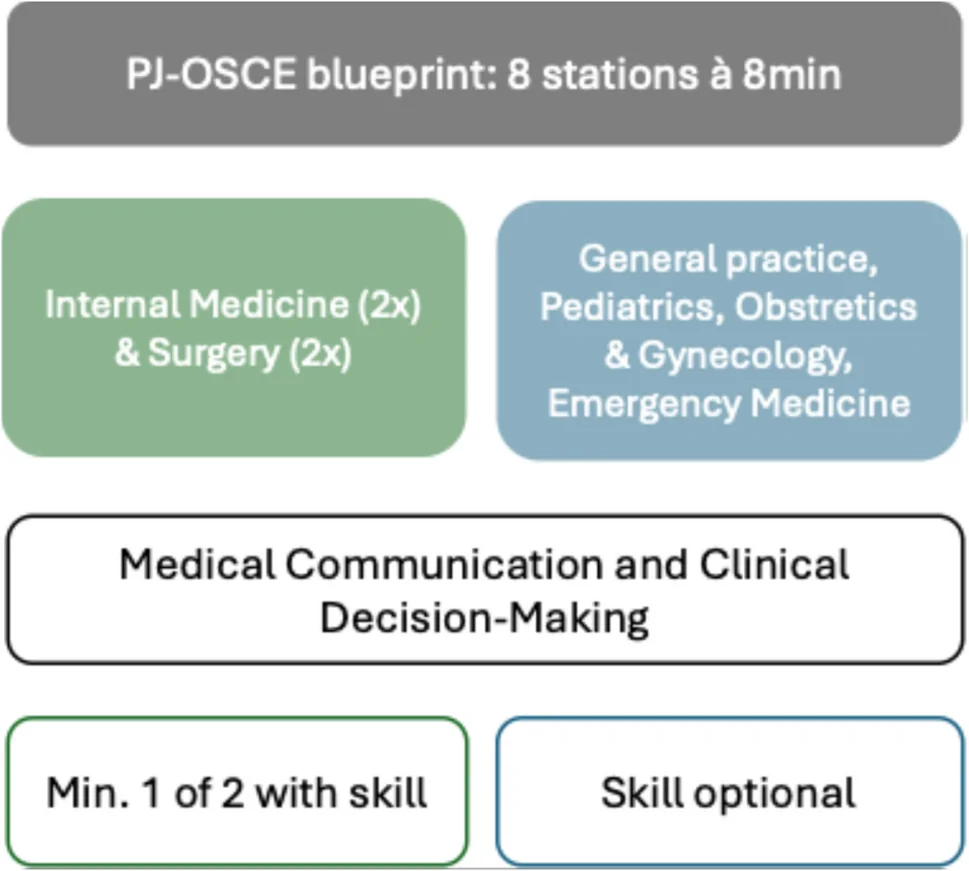
Blueprint of the PJ-OSCE by clinical discipline. Each examination circuit consisted of eight different stations: two stations each from surgery and internal medicine, including one skills-based station per discipline. One station each was drawn from general practice, pediatrics, obstetrics & gynecology, and intensive care/emergency medicine. All stations included assessment of clinical decision making and physician–patient and/or interprofessional communication

Beginning with the 2023/24 academic year, the PJ-OSCE was formally integrated into the curriculum and linked to five clinical clerkships/modules (general practice, surgery, gynecology, internal medicine, and pediatrics) of the fourth and fifth years of study. This integration was discussed and approved by the undergraduate medical education committee and is reflected in the examination blueprint (Fig. 1).

Additional to digital information on the PJ-OSCE, a peer-led practice day was offered to support skills training and simulated OSCE station practice. Standardized simulated patients received specialized role-specific training in preparation for the examination.

The PJ-OSCE was conducted over eight days in June 2024 at the MHH SkillsLAB and consisted of eight stations of 8 min each, with 2-minute transition intervals. Circuit student numbers varied slightly (*n* = 7–24) due to sequential filling of morning/afternoon sessions and individual student absences on the exam day. Examiners completed standardized assessment rubrics in real time using dedicated examination software (Umbrella Consortium for Assessment Networks, Institut für Kommunikations- und Prüfungsforschung gGmbH (UCAN), tOSCE App, Version 1.14.11). The tOSCE App is part of the UCAN platform and is used at medical faculties; it enables examiners to complete standardized assessment rubrics digitally in real time, automatically calculates scores per station and circuit, and exports data for psychometric analysis. For quality assurance and further development, students and examiners were invited to complete an anonymous, voluntary online questionnaire after completing the OSCE. The questionnaire included items assessing overall examination quality on a 15-point scale (0 = very poor, 15 = excellent), perceived learning gain, and evaluation of the examination format using a 6-point Likert scale.

### Ethics & statistics

The evaluation of the educational program received ethical approval from the Ethics Committee of Hannover Medical School (approval number 11455_BO_K_2024). All procedures were performed in accordance with the Declaration of Helsinki. We used Microsoft Excel (Office LTSC Professional Plus 2024) for statistics. Test scores and evaluation scores are reported with mean and standard deviation (SD). Circuit-level reliability was reported with Cronbach’s alpha. Item difficulty was determined as the proportion of students achieving the maximum score per checklist item. For evaluation, the questionnaires were created via ScoSci Survey (program version 3.7.06) and distributed online. Participants stated digital informed consent prior to participation.

## Results

### Development of the PJ-OSCE

The project team consisted of five members. A total of 34 PJ-OSCE stations were developed by 17 (specialist) physicians and five students under supervision, using the previously developed station templates. The primary focus during station development was on defining specific learning objectives and tasks related to clinical decision-making. Each station included at least one subtask dedicated to communication with either patients, relatives or members of the healthcare team. Of the 34 developed stations, 82.3% (28/34) included elements of clinical decision-making and 76.5% (26/34) contained a skill. Four stations were developed by students under supervision.

The stations were not limited to single disciplines but also addressed interdisciplinary cross-sectional topics, such as a station on suspected child abuse developed collaboratively by pediatrics and forensic medicine. Following an initial review by the PJ-OSCE team, a total of 20 interdisciplinary workshops were conducted.

Each station consisted of four to five individual tasks with different weightings, resulting in a total of 100 points per station. Differential weighting was intentionally designed to prioritize clinically critical tasks within each station, ensuring that students could not pass by accumulating points on less essential sub-items while failing to demonstrate key competencies. Pass–fail cut scores determined through interdisciplinary standard setting using the modified Angoff method ranged between 55.0% and 59.0% (Table 2).

### Feasibility assessment and pilot testing

The first pilot examination was conducted in August 2022 and comprised nine stations (maximum score: 900 points) covering neurology, pediatrics, surgery, general practice, emergency medicine, internal medicine, public health, and gynecology. Nine volunteer students from the fifth and sixth years of study participated. A total of 22 individuals were involved in the pilot (nine examiners, nine standardized patients, one overall coordinator, and three examination supervisors). Despite the considerable staffing requirements, the project team rated the PJ-OSCE as feasible.

A second pilot examination was conducted in October 2022 following minor revisions based on feedback from the first pilot. Key revisions included: adjustment of station timing (reduction from 10 to 8 min based on observed student completion rates), clarification of task instructions that had been identified as ambiguous during the first pilot, and refinement of two scoring rubrics where inter-rater variance had been observed. Three new stations were added to broaden domain coverage. This second pilot consisted of 12 stations and included 23 volunteer students from the fifth and sixth years of study. Across both pilot runs, the mean student score was 766.91 points (63.9%).

Faculty stakeholders from clinical departments and administration who attended the publicly conducted pilot examinations also evaluated the PJ-OSCE positively. In all four student focus groups, participants emphasized the overall relevance of the examination and its close alignment with clinical practice. However, time constraints during the OSCE and the associated stress were consistently identified as negative aspects. In three of the four focus groups, students also criticized an unclear performance expectation and expressed a preference for using OSCEs as formative learning tools rather than summative assessment formats.

### Curricular implementation and examination delivery

Following successful pilot testing, the PJ-OSCE was formally integrated into the curriculum of the undergraduate medical program. The study commission approved a concept linking the PJ-OSCE to the five mandatory clinical clerkships specified in the Medical Licensure Regulations (general practice, surgery, gynecology, pediatrics, and internal medicine). During a transition phase (2023–2025), the PJ-OSCE was mandatory but ungraded. Beginning with the 2025/26 academic year, the PJ-OSCE was established as a summative examination format, replacing the individual final examinations in the respective clerkships/modules.

Prior to the PJ-OSCE in 2024, a voluntary practice day was offered at the MHH SkillsLAB, during which 117 students participated in peer-teaching sessions to practice and refine their clinical skills and competencies.

The first mandatory PJ-OSCE examinations were conducted in June and August 2024 and were organized by a team of 17 individuals, including members of the PJ-OSCE team, SkillsLAB staff, and the standardized patient program. 81 individuals, who had received prior digital or in-person training, served as examiners across 120 examination sessions, corresponding to approximately 520 examiner-hours in total. A total of 173 standardized patient slots were filled by 63 trained individuals across all stations; this corresponds to approximately 750 SP-hours in total. In total, 278 students participated in the examination, yielding an examiner-to-student ratio of approximately 1:2.3 and an SP-to-student ratio of approximately 1:1.6 per examination day.

Based on the experience and evaluation from the pilot OSCEs, each of the 15 differently composed examination circuits consisted of eight stations of 8 min each, with 2-minute transition times, resulting in a total circuit duration of 78 min. The number of eight stations was determined by balancing psychometric requirements (literature supports ≥ 6–8 stations for adequate reliability in this format), domain coverage across five contributing clerkships, logistical feasibility within the MHH SkillsLAB infrastructure, and total examination duration [5]. Two cohorts were examined in the morning and three cohorts in the afternoon, resulting in a total examination duration of 7.5 days, including retests.

A maximum of 800 points could be achieved per circuit. In accordance with a summative OSCE format, the pass–fail cut scores established through standard setting ranged from 440 (55.0%) to 472 (59.0%) points. With one exception, the calculated reliability (Cronbach’s alpha) of the circuits exceeded 0.80 (mean 0.84, SD 0.06). The lower coefficient in Circuit 3 (α = 0.68) reflects ceiling effects in leniently rated communication items and a narrow spread of candidate ability within this circuit rather than a deficiency of the station blueprint. Examination difficulty ranged from 0.71 to 0.79 (mean 0.75, SD 0.03) (Table 2).

The mean score achieved by students was 603.08 points (SD 67.57). Eight students (2.9%) did not reach the pass threshold (Table 2). The reduced time of 8 min per station did not result in lower completion rates for the skills-based tasks compared to the pilot OSCE.

**Table 2:** Overview of examination characteristics of the PJ-OSCE 2024

Circuit	Cronbach’s alpha	Examination difficulty	Mean score achieved*		Maximum score achieved*		minimum score achieved*		Pass mark		Students passed		Students failed	
	α	*P*	M (SD)	% (SD)	*n*	%	*n*	%	*n*	%	*n*	%	*N*	%
1	0.81	0.77	611.88 (61.22)	76.5 (7.65)	702	87.8	504	63.0	456	57.0	16	100.0	0	0.0
2	0.83	0.79	631.48 (57.83)	78.9 (7.23)	702	87.8	526	65.8	456	57.0	23	100.0	0	0.0
3	0.68	0.73	581.94 (38.91)	72.7 (4.86)	637	79.6	510	63.8	440	55.0	16	100.0	0	0.0
4	0.85	0.77	609.00 (58.73)	76.1 (7.34)	696	87.0	471	58.9	440	55.0	23	100.0	0	0.0
5	0.86	0.78	614.20 (70.58)	76.8 (8.82)	698	87.3	484	60.5	440	55.0	15	100.0	0	0.0
6	0.81	0.77	618.58 (60.41)	77.3 (7.55)	734	91.8	479	59.9	448	56.0	24	100.0	0	0.0
7	0.80	0.76	601.40 (56.91)	75.2 (7.11)	673	84.1	431	53.9	472	59.0	14	93.3	1	6.7
8	0.93	0.72	575.18 (98.09)	71.9 (12.26)	687	85.9	378	47.3	464	58.0	19	86.4	3	13.6
9	0.85	0.75	603.25 (59.33)	75.4 (7.42)	701	87.6	483	60.4	464	58.0	16	100.0	0	0.0
10	0.94	0.76	606.86 (86.62)	75.9 (10.83)	735	91.9	341	42.6	448	56.0	21	95.5	1	4.5
11	0.88	0.78	619.69 (75.77)	77.5 (9.47)	704	88.0	409	51.1	456	57.0	15	93.8	1	6.2
12	0.80	0.77	622.22 (55.86)	77.8 (6.98)	717	89.6	524	65.5	440	55.0	23	100.0	0	0.0
13	0.82	0.71	568.94 (59.63)	71.1 (7.45)	665	83.1	459	57.4	448	56.0	16	100.0	0	0.0
14	0.85	0.72	581.33 (61.00)	72.7 (7.63)	682	85.3	374	46.8	456	57.0	23	95.8	1	4.17
15	0.82	0.71	572.29 (69.26)	71.5 (8.66)	667	83.4	437	54.6	456	57.0	6	85.7	1	14.3
Overall	0.84	0.75	603.08 (67.57)	75.2 (8.61)							270	97.1	8	2.9

*Maximum achievable score per examination: 800 points (8 stations with 100 points each). Reliability assessed using Cronbach’s alpha (threshold ≥ 0.80 for high-stakes assessments). Examination difficulty reported as facility index (proportion of maximum score achieved). Maximum, minimum, and mean achieved scores, as well as individual pass thresholds and corresponding numbers of students passing and failing, are reported for 15 examination circuits

A total of 245 students participated in the evaluation, with 224 fully completed questionnaires. On a 15-point grading scale, students rated the PJ-OSCE with a mean score of 10.90/15 points (SD 2.54; *n* = 225). 41% of students reported a large or very large subjective learning gain. Overall, 123 students (50.2%) reported high to very high levels of enjoyment during the PJ-OSCE, 78 students (31.8%) responded neutrally, and 36 students (14.7%) reported little or very little enjoyment (including nonresponses).

Among examiners (*n* = 51), the mean overall rating was 12.59/15 points (SD 1.67). The examination format was rated as appropriate by 86.3% of examiners (*n* = 44), while 1.9% (*n* = 1) rated it as inappropriate; 11.8% (*n* = 6) did not respond to this item.

## Discussion

The development of the summative PJ-OSCE at Hannover Medical School (MHH) was based on established concepts and quality-assured templates [9]. This approach enabled the rapid design of 34 OSCE stations by subject-matter experts, their testing in workshops, and the definition of pass–fail cut scores using standardized standard-setting procedures. The involvement of students in this process proved valuable, as it allowed learner-specific needs and perspectives to be considered during the design phase [10]. Complex OSCE stations have been developed in other contexts, such as assessing ethical decision-making competencies in nursing education [11]. These experiences supported the use of integrated scenarios in the PJ-OSCE that simultaneously assess communication skills, examination techniques, and clinical decision-making. Through the collaboration of specialist physicians, educational researchers, and experts in medical education, realistic clinical cases were successfully translated into standardized assessment rubrics [12].

The primary purpose of the pilot examinations was to assess the feasibility of implementing a complex summative assessment [13]. Similar approaches have been proven useful in other settings to derive “lessons learned” for subsequent implementation [12]. Despite the anticipated high demands on time and personnel resources [14], the PJ-OSCE at MHH was evaluated as feasible by the project team, examiners, and students. To contextualise the resource burden: the PJ-OSCE 2024 required 81 trained examiners, 63 standardised patients filling 173 SP slots, and a coordination team to deliver 120 examination sessions over 7.5 days. Quantification of full cost-effectiveness, including overhead and opportunity costs, remains a priority for future work. The high overall pass rate (97.1%) warrants critical reflection. Possible explanations include: that the examination was appropriately calibrated for students who had completed all required clinical clerkships, that Angoff cut scores were set conservatively by the standard-setting panel, and/or that the transition phase with a mandatory and ungraded OSCE may have sensitized students and faculty to the examination format. Longitudinal monitoring of pass rates and station-level performance will be essential as the PJ-OSCE transitions to a fully graded summative assessment. Stakeholders were actively involved during the pilot phase to support effective change management. Feedback obtained from student focus groups was incorporated into subsequent revisions of the concept and examination blueprint, ultimately resulting in an OSCE format consisting of eight stations of 8 min each.

Previous formative OSCE implementations in German medical education have demonstrated considerable deficits in practical clinical skills among final-year students, supporting the rationale for summative assessment at this stage [15].The curricular integration of the PJ-OSCE into the disciplines and clerkships of internal medicine, surgery, gynecology, pediatrics, and general practice was achieved with the involvement of all teaching coordinators and the Office of the Dean of Studies. A comparable “PJ readiness OSCE” was introduced at the University of Würzburg in 2019/20 and similarly linked to clinical clerkships. From a curriculum development perspective, a mandatory OSCE appears beneficial, as it intentionally guides student learning and places greater emphasis on practicing clinical skills [16]. The high participation rates in the voluntary practice day suggest that peer-teaching–based practical training is a preferred preparatory approach among students.

The implementation of the PJ-OSCE required the involvement of many examiners and standardized patients. The reliability and validity of complex assessments depend on numerous human and contextual factors, including the examination setting, motivation, and the gender of students, standardized patients, and examiners [17]. The structured training of examiners and standardized patients partly conducted digitally, along with standardized assessment rubrics and a clearly defined examination blueprint contributed to the high reliability and balanced difficulty of the PJ-OSCE circuits. Comparable results across morning and afternoon cohorts further suggest that information exchange between cohorts was unlikely or did not measurably affect scoring outcomes.

During the transition phase, the OSCE was a mandatory curricular requirement without grading, although it was designed as a summative assessment from the outset. This approach allowed students to familiarize themselves with a new examination format while enabling the institution to gain organizational experience in administering an OSCE to an entire cohort of approximately 320 students. The evaluation results indicated that students recognized the relevance of the assessment and reported perceived learning gains, while examiners regarded the OSCE as an appropriate examination format for the assessed competencies.

Overall, the process demonstrated that the successfully designed, piloted, and curricularly implemented PJ-OSCE at MHH can function as a summative assessment of practical skills and Entrustable Professional Activities prior to entry into the Practical Year. Nevertheless, the extent to which this supports students in further developing practical skills in the PJ (in comparison to previous types of examinations) requires further research.

Initially, the development of the PJ-OSCE was motivated by a proposed revision of the Medical Licensure Regulations [3]. The absence of external regulatory pressure represents a limitation, particularly for change management processes. Additional challenges include the narrow examination time window between completion of all clinical clerkships and preparation for the second national medical examination, as well as the temporal gap between clerkship completion and the OSCE itself. Several limitations of the present study should be acknowledged. First, this is a single-institution, descriptive feasibility study without a comparator group [13]; causal claims about the effect of the PJ-OSCE on student competence cannot be made on this basis. Second, evaluation data rely substantially on self-reported evaluations from students and examiners, which are subject to response bias. Third, as pass/fail cut scores were established using a criterion-referenced method (modified Angoff), the observed pass rate reflects performance relative to predefined competency standards rather than a targeted failure quota; nonetheless, the high pass rate (97.1%) may limit discriminatory power in its current form and warrants longitudinal monitoring across future cohorts. Fourth, the generalizability of the findings may be limited by the specific curricular context of MHH (HannibaL model curriculum) and the German medical licensing framework. Fifth, a comprehensive cost analysis was beyond the scope of this study; future work should quantify the full resource burden to support evidence-based implementation decisions at other institutions. A formal validity study using Kane’s argument-based approach is proposed as an important next step to strengthen the evidence base for the PJ-OSCE.

Beginning with the 2025/26 academic year, the PJ-OSCE will be fully established as a summative examination at MHH. To maintain formative elements, various feedback instruments will be evaluated concurrently. The high participation in the voluntary practice day suggests the need for practical (peer-led) skill training for preparation of the OSCE. Accordingly, curricular adjustments with a stronger focus on practical skills, guided by principles of constructive alignment, are planned and will be scientifically evaluated. The frequency and timing of such practice opportunities — ideally embedded earlier in the curriculum rather than immediately preceding the examination — represent an area for ongoing development and will be informed by student feedback and learning outcome data in future cohorts. The transition from undergraduate to postgraduate training represents a critical junction at which practical skill deficits become particularly consequential; vertically integrated curricula have been shown to improve readiness for practice at this stage [18]. The PJ-OSCE at MHH, embedded within the HannibaL model curriculum, directly addresses this transition point. Overall, the PJ-OSCE developed at MHH offers a validated blueprint that may serve as a valuable reference for other medical faculties seeking to implement a combined summative and formative OSCE prior to the Practical Year.

## Data Availability

The datasets generated and/or analysed during the current study are not publicly available due to ongoing evaluation of the OSCE but are available from the corresponding author on reasonable request.

## Abbreviations

EPA: Entrustable professional activities
MHH: Hannover Medical School
NKLM: German National Competence-Based Learning Objectives Catalogue
OSCE: Objective structured clinical examination
PJ: Practical year (Praktisches Jahr)
SD: Standard deviation
